# Executive function in alcohol use disorder with low psychiatric comorbidity: Comparison with a non-clinical sample and predictive value for treatment outcome

**DOI:** 10.1371/journal.pone.0350955

**Published:** 2026-09-11

**Authors:** Stina Ingesson-Hammarberg, Maria Å. Garke, Lotfi Khemiri, Anders Hammarberg, Nitya Jayaram-Lindström

**Affiliations:** 1 Centre for Psychiatry Research, Department of Clinical Neuroscience, Karolinska Institutet, & Stockholm Health Care Services, Region Stockholm, Sweden; 2 Department of Medical Epidemiology and Biostatistics, Karolinska Institutet, Stockholm, Sweden; Jawaharlal Institute of Postgraduate Medical Education and Research, INDIA

## Abstract

**Background:**

Executive functions (EF) encompass abilities such as planning, decision-making, and inhibitory control, critical for learning, establishing and maintaining behavioral change. The association between alcohol use disorder (AUD) and impairments in EF are well established. However, prior research is dominated by studies on convenience samples including individuals with severe AUD with high levels of psychiatric comorbidity, which limits generalizability. The present study therefore aimed to investigate the degree of impairment and predictive ability of EF, on alcohol consumption, among individuals with moderate AUD with low levels of psychiatric comorbidity.

**Methods:**

Adults with moderate AUD (*n* = 147) were recruited at three specialized addiction outpatient clinics in Stockholm, to a randomized controlled trial investigating the efficacy of two psychological treatments. Participants underwent neuropsychological testing before treatment. Eight tests from the CANTAB^®^ battery were administered at baseline, assessing mental flexibility, sustained attention, visuospatial working memory, response inhibition, and delay discounting. Assessments of alcohol use and related symptoms were conducted at baseline, the 12- and 26-weeks follow-up. A non-clinical reference sample (*n* = 72) completed corresponding CANTAB^®^ tests. The two groups were compared regarding EF using descriptive statistics and *t*-tests, and the predictive value of EF for reduction in alcohol consumption, was investigated using multiple regression models.

**Results:**

Individuals with AUD did not perform worse on any of the tests on executive function (CANTAB^®^) as compared to the non-clinical reference sample. Measures of EF were not significant predictors for reduction in alcohol use for the 12-week, or the 26-week follow-up.

**Conclusions:**

EFs were not impaired and were not a clinically relevant predictor of treatment outcomes in this population with AUD. Future research on EF as a predictor in AUD treatment, needs to corroborate the present findings, and include other populations, e.g., with different socio-economic backgrounds and by including other methodologies for measuring EF.

## Introduction

Alcohol use disorder (AUD) is a psychiatric disorder characterized by impaired control over alcohol consumption, as well as negative social, physical, and psychological consequences of alcohol use, and is associated with substantial mortality and morbidity [1]. Although not included as a diagnostic criterion in the Diagnostic and Statistical Manual of Mental Disorders (DSM-5) [2], repeated findings have shown that treatment-seeking individuals with AUD often exhibit cognitive impairments that can hamper recovery [3–6]. Cognitive dysfunction in AUD involves impairments in areas such as attention, processing speed, learning, motor-, visuospatial- and memory functions [3], as well as a more extensive negative impact on overall levels of executive functioning (EF) [7–9]. EF is involved in the regulation of goal-directed behavior, such as planning, decision-making, self-monitoring, and inhibitory control [5], also suggested to be specifically related to alcohol misuse [5,9,10].

There is a growing body of research investigating EF impairments in individuals with AUD, and its effect on treatment outcomes [4,11–13]. Impulsivity, which may be defined as a predisposition toward rapid, unplanned reactions without regard to potential negative consequences, is a construct strongly associated with psychiatric- and substance use disorders (SUD) [9,10]. EF and impulsivity have been suggested to be interconnected, as being the two antipodes of a continuum [14]. Impulsivity is a heterogeneous construct and involves several subconstructs; positive and negative urgency, response- and reflection impulsivity, and delay discounting [15]. Among these higher-order functions, specific abilities have been pointed out as potential predictors of treatment outcomes [13]. For example, response inhibition and delay discounting have been proposed as predictors of both retention and outcome in the treatment for SUD [12,16,17]. More specifically, response inhibition (meaning the ability to inhibit a response) is associated with both relapse and treatment completion [17]. These abilities are measured with go/no-go-tasks (GNG) or the Stop Signal Task (SST). Further, delay discounting, as measured by the Information Sampling Task or the Cambridge Gambling Task [18], assesses the preference of an immediate smaller reward and the choice of a delayed greater reward. Delay discounting is associated with the development of SUDs and less successful treatment outcomes [10,18]. For example, the preference for short-term high rewards and the risk of high losses, as compared to lower rewards and lower risk, is more common in abstinent alcohol-dependent individuals, than in healthy individuals [19,20].

Taken together, the association between AUD and EF impairments is well established, but less is known to what extent impairments are prevalent in individuals with AUD in general, and in individuals with mild to moderate AUD [3,9].The literature on cognitive dysfunction in AUD is dominated by studies in recently detoxified inpatients, often categorized as being in chronic states of AUD and with high levels of psychiatric comorbidities [3,9,21]. Other suggested limitations are over-representation of convenience samples and male participants [21]. Treatment naïve-patients, females, and individuals with low levels of psychiatric comorbidities have not been sufficiently investigated regarding impairments of EF in AUD. Lastly, it is not known if impairments in EF are clinically relevant predictors of treatment outcomes in patients with AUD in general. In summary, the mentioned limitations, hampers the generalizability of the findings to the broader population of individuals with AUD.

The aim of the current study was twofold. First, to the aim was to investigate if adult treatment-seeking individuals, with a treatment goal of controlled drinking, have identifiable impairments in EF compared to a non-clinical reference sample of individuals without alcohol problems. The hypothesis was that individuals with AUD would perform significantly worse on tests measuring EF. More specifically, we hypothesized that indices of impulsivity, including response inhibition and delay discounting would be significantly different compared to the non-clinical reference sample. Second, the aim was to investigate if specific measures of EF were identifiable as predictors of treatment outcomes in individuals with AUD, undergoing psychological treatment with a goal of controlled drinking. We hypothesized that the indices of response inhibition and delay discounting (Stop Signal Task and Information Sampling Task) would predict outcomes of mean weekly alcohol consumption and proportion of days with drinking.

## Methods

### Participants and procedure

#### Sample with AUD.

Data was included from a randomized controlled trial including 250 individuals with AUD, which aimed to investigate if Behavioral Self-Control Training (BSCT) was superior to Motivational Enhancement Therapy in reducing alcohol consumption. The primary outcome of the main study was the difference in weekly alcohol consumption between groups at 26 weeks post inclusion. The study was conducted at three addiction outpatient clinics at Stockholm Centre for Dependency Disorders, Sweden. For full details of the main clinical trial, see Ingesson-Hammarberg [22]. The study sample consisted of patients newly admitted to three study clinics and were recruited either by self-referrals or via social media advertisement. At their first assessment meeting in the clinic, patients were asked if they wanted to participate in a trial comparing two treatments for controlled drinking. Eligible participants were adults between 18−70 years, fulfilling a diagnosis of AUD according to the DSM-5, with a stated treatment goal of controlled drinking. The exclusion criteria were: fulfillment of any other SUD except nicotine use disorder, a severe psychiatric condition (suicidal ideation, severe major depression, untreated bipolar disorder, psychotic disorder), on antidepressant medication for less than three months, any severe adverse event related to alcohol consumption during the last 12 months (such as intoxication resulting in hospital care, requiring pharmacological treatment for withdrawal symptoms, delirium tremens, or significantly elevated liver enzymes [three times the clinical cutoff]).

Patients were scheduled for an assessment interview at the clinic and screened for eligibility according to a standardized protocol by trained assessors (n = 5). All participants gave oral and written consent to participate in the study. After being included, and no later than one week after inclusion, all participants performed the cognitive testing. The neuropsychological computerized test battery CANTAB^®^ [23] used to measure EF, was administered in a silent testing room by trained assessors. All participants took a breathalyzer test before the testing session, to rule out current intoxication. The session lasted between 1.25 to 2 hours. All participants were allowed to use nicotine and to drink coffee before the session. After the cognitive assessment, all participants commenced treatment. The main study was approved by the Swedish Ethics Review Authority in Stockholm (2016/634–31/2) and registered with ISRCTN, trial no. 05/06/2018.

The original study design included cognitive testing at baseline for all 250 participants, but the trial design was changed in March 2020 due to the COVID-19 pandemic. Between 20^th^ of March and until finalization of recruitment for the study in December 2020, the included participants no longer performed cognitive testing, due to the risk of infection for participants and clinical staff. The psychological treatment during this phase was conducted via video sessions. The final sample size in the current study, given the change in trial design, therefore comprised 147 individuals who completed the CANTAB^®^.

### Non-clinical reference sample

The non-clinical sample was used as a reference group with regards to CANTAB^®^ outcomes in the current study. This sample consisted of 90 participants who were recruited in another study evaluating the effect of AUD family history on cognition [24]. Advertisements in social media were used to recruit the participants. The inclusion criteria were 18−60 years of age and the main exclusion criteria were the fulfillment of any substance abuse or dependence (except nicotine) diagnosis according to the DSM-IV, severe major psychiatric disorder (e.g., psychotic disorders, bipolar disorder) or family history of these disorders, and use of narcotics or positive breathalyzer test before performing the testing. Further exclusion criteria were applied for the non-clinical reference sample which were not applied to the sample with AUD, such as regular use of psychoactive medications (e.g., antipsychotic, antiepileptic, or opioid pain medications) during the last 12 months (except antidepressant medication), a medical history of stroke, intracranial hemorrhage, or severe head trauma. The study was approved by the Swedish Ethics Review Authority in Stockholm (2016/1113–312).

### Measures

#### Diagnostic assessment.

All participants in the current study, i.e., the sample of individuals with AUD as well as the non-clinical sample, were assessed at baseline and underwent a structured diagnostic interview to screen for AUD and other psychiatric diagnoses using the Mini International Neuropsychiatric Interview [25].

### Computerized tasks

The neuropsychological test battery comprised several computerized tasks of cognitive function from the neuropsychological test battery CANTAB^®^ (www.camcog.org) developed by Cambridge University [23]. The test battery that was chosen for this study contained eight tests and was chosen to assess different aspects of EF, such as response inhibition, flexibility in attention shifting, working memory, and decision making, based on the recommendations from Cambridge Cognition. The different targets for the specific tests are described below. A touch screen tablet PC (MOTION model J3500-i7B) and a press pad were used for all test sessions, provided by Cambridge Cognition Ltd. The following tests were included in the test battery: 1) Spatial Working Memory task (SWM) assessing the subject’s ability to retain spatial information (visuospatial working memory) and strategy used in the search for a hidden token. The main outcomes for this test are Between errors and Strategy. 2) Stop signal task is a stop signal response inhibition test, in which the subject is instructed to either press or inhibit the response to press when being instructed to. The main outcome of this test is the Stop Signal Reaction Time (SSRT). 3) Stockings of Cambridge (SOC) is measuring spatial planning, strategy, and the ability to retain visual information. The outcomes are a) Problems solved in minimum moves, b) Initial thinking time, c) Subsequent thinking time, and d) mean moves for the most difficult 5-move problems. 4) Intra/Extradimensional shift (IED) is a test of rule acquisition, shifting, and flexibility of attention when the stimuli is changing. The main outcomes are a) the number of errors made, b) the number of trials, and c) number of stages completed. 5) Information Sampling Task (IST) is a task designed to measure pre-decisional processing and delay discounting, i.e., attribution of the value of an immediate reward vs a delayed reward (da Matta et al., 2012b). The main outcomes of the test are a) Discrimination errors, b) Mean P (correct), c) Sampling errors, and d) The mean number of boxes opened per trial across both win conditions. 6) Rapid Visual Processing task (RVP) is a task measuring sustained attention when observing and detecting specific digit sequences. The two main outcomes are Probability of hit and “P Probability”. 7) Attention Switching Task (AST) involves a test that involve conflicting instructions, and tests the abilities of information processing, sustained attention, and mental flexibility. The main outcomes are Median Switching cost and Median congruency cost. 8) In the Cambridge Gambling task (CGT) the subject is instructed to decide to bet a certain value on their decision, which assesses decision-making and risk-taking behavior. The main outcomes are risk adjustment and delay aversion.

### Self-report measures

Symptoms of attention-deficit and hyperactivity disorder (ADHD) were measured using the ADHD-Adult Self Report Scale [26].

### Outcomes in predictor models

The following variables were chosen as outcomes for the prediction models in the sample with AUD, measured at baseline, 12-, and 26 weeks post inclusion:

*Mean weekly alcohol consumption.* Mean weekly alcohol consumption was calculated as the mean number of standard drinks per week during the preceding 90 days (12.86 weeks), based on the Timeline Follow-Back method of 90 days (TLFB) [27] for the registration of number of standard drinks (12 grams of pure ethanol) consumed per day.

*Proportion of days with drinking.* The proportion of days with drinking during the preceding 90 days was calculated by dividing the number of days with drinking by 90. This outcome was also based on the TLFB.

*Carbohydrate-deficient transferrin.* Carbohydrate-deficient transferrin (CDT) is a biological marker of alcohol consumption that was assessed from blood tests.

*Alcohol Use Disorders Identification Test*. The Alcohol Use Disorders Identification Test (AUDIT) [28] total score was used as a measure of the severity of alcohol problems.

### Interventions

The sample of patients with AUD received either 1) treatment based on BSCT, which is a manual-based cognitive behavioral treatment [29,30] consisting of five sessions, including self-monitoring of alcohol consumption, identification of risk situations, moderation- and relapse prevention strategies; or 2) MET, which is a four-session manual-based treatment, including assessment and personalized feedback, and three following Motivational Interviewing sessions [31,32]. For further information on these interventions, see the published manuscript of the original trial [22].

### Data preparation and statistical analyses

Data preparation and statistical analyses were performed in R version 4.1.3 [33], see Supplementary material for package details. The data analysis plan was pre-registered before analyses were commenced (see https://doi.org/10.17605/OSF.IO/D35U4). Inference criteria included an alpha level of  .05, with two-tailed tests (to account for the possibility that the effect is in the opposite direction than what was expected), as well as reporting of all complete *p*-values for all tests and reporting of bootstrapped confidence intervals. For the complete case analyses, participants with data on all CANTAB^®^ measures were included (*n* = 147 patients with AUD and *n* = 72 non-clinical participants). Five cases of outliers were identified upon inspection of the CANTAB^®^ measures, and these were deemed valid in the sense that they did not occur due to technical failure or coding error. According to the plan, analyses were run both with and without the inclusion of these cases. Since no meaningful differences in results were found, all analyses were reported including these cases. Missing data was identified primarily on the CANTAB^®^ tests. There were two main reasons for this data loss; 1) the premature ending of testing in the main trial due to the COVID-19 pandemic (86%); 2) technical failure while testing, or participants wishing to prematurely discontinue a test (14%). Due to the nature of the missing data in the CANTAB^®^ measures being mainly systematic and could be defined as missing at random [34], data in the current study was deemed appropriate for imputation as a robustness check. This was done using multiple imputation with predictive mean matching [35].

Summary statistics were derived for the available demographic variables collected at baseline. Potential differences in demographic variables (age, gender, educational level) between patients with AUD and the non-clinical reference sample were assessed using *t*-tests and chi-squared tests as appropriate. To investigate between-group differences (patients with AUD and non-clinical sample) on the cognitive measures (CANTAB^®^) conducted at baseline, descriptive statistics (mean and standard deviations), effect sizes (Cohen’s *d*), and regression models were used. The regression models were multiple linear models, specified with the CANTAB^®^ test set as outcome and group status (patient with AUD or non-clinical) as independent variable. Age and educational level were included as covariates to adjust for the influence of these variables given the existing differences between groups.

The predictive value of cognitive performance on treatment outcomes was assessed in a series of multiple linear regression models. A separate model was fitted for each outcome (TLFB mean weekly alcohol consumption and proportion of days with drinking) at each follow-up in a cross-sectional manner, with the 26-week follow-up chosen as the primary endpoint (as in the main parent study RCT). As the 12- and 26-week assessments correspond to distinct, pre-specified endpoints (as per the parent study RCT), each follow-up was modelled separately rather than as repeated measurements. The outcomes CDT and AUDIT total score were analyzed in the same manner, for exploratory purposes. TLFB mean weekly alcohol consumption and CDT were square-root transformed to address positive skew. In each model, the predictor of interest was the recommended primary index for the respective CANTAB^®^ test (one index for SST, four indices for IST, each entered in a separate model). Models adjusted for the baseline value of the outcome, gender, age, baseline impulsivity (ASRS), and treatment arm (BSCT vs. MET). This approach replaced the pre-registered analysis, which could not be fitted as specified because the planned model was overly complex for the available data. Multicollinearity was ruled out by assessing the Variance Inflation Factor. Effect sized were quantified as squared semi-partial correlations, representing the unique variance explained by each predictor. To control the false discovery rate across all tests (excluding exploratory analyses), *p*-values were adjusted using the Benjamini-Yekutieli procedure for dependent tests [36].

## Results

A summary of demographic characteristics and clinical variables are presented in Table 1. Participants in the patient sample had, on average, a moderate level of AUD according to the DSM-5, were 51 years old (*m* = 51.97, *sd* = 11.47), well-educated, and 46% were women (see Table 1 for more detail).

**Table 1:** Demographic information and descriptive statistics across the sample with AUD and non-clinical reference sample reported at baseline (all available data).

Variables	AUD (*n* = 147)	NCS (*n* = 72)
	*n* (%)	*n* (%)
*Gender*		
Female	68 (46)	38 (53)
Male	79 (54)	34 (47)
Education level*		
No basic educational level	1 (1)	1 (1)
Secondary school	7 (5)	4 (6)
Upper secondary school	39 (27)	34 (47)
University	99 (67)	33 (46)
Family history of AUD (sibling and/or parent; *n* = 138)	89 (64)	–
Major Depressive Disorder	9 (6)	–
Generalized Anxiety Disorder	12 (8)	–
Post-Traumatic Stress Disorder	3 (2)	–
Attention-Deficit Hyperactivity Disorder	9 (6)	–
Autism Spectrum Disorder	10 (7)	–
Lifetime fulfillment of a manic episode, hypomania	0 (0)	–
Bipolar disorder (any)	0 (0)	–
Lifetime fulfillment of any psychotic disorder	0 (0)	–
Nicotine use (cigarette, snuff, nicotine replacement)	54 (37)	–
	*Mdn (IQR)*	*Mdn (IQR)*
Number of endorsed AUD DSM-5 criteria	5 []3 [–]6 []	–
Mean weekly alcohol consumption (standard drinks)	19 []15 [–]27 []	2 (0-3)
	*M (SD)*	*M (SD)*
Age*	51.97 (11.47)	47.58 (11.91)
Proportion of days with drinking	0.68 (0.22)	0.15 (0.13)
Proportion of days with heavy drinking	0.40 (0.33)	0.01 (0.03)
AUDIT total score (*n* = 146)	18.00 (5.80)	–
AUDIT C total score (*n* = 146)	7.94 (1.77)	2.13 (1.55)
SIP total score (*n* = 144)	11.01 (6.23)	–
ASRS total score (*n* = 144, *n* = 59)	20.13 (11.85)	16.98 (9.1)
CDT (*n* = 144)	1.97 (1.33)	–

*Note*. AUD = Alcohol Use Disorder, NCS = Non-Clinical Reference Sample, TLFB = Timeline Follow-Back, AUDIT = Alcohol Use Disorder Identification Test, SIP = Short Index of Problems, ASRS = Adult ADHD Self-Report Scale, CDT = Carbohydrate Deficient Transferrin. Asterisk (*) denotes a statistically significant difference between groups.

There was a statistically significant difference in age and educational level between the groups. The non-clinical reference sample were slightly younger on average, and had a smaller proportion of participants having completed university education. Contrary to the hypothesis, between-group analyses revealed no significant differences in executive function performance between patients with AUD and the non-clinical reference sample.”(see Table 2). Hence, there were no detectable impairments in EF when the AUD sample was compared to a non-clinical reference sample. However, one specific EF measure, the AST congruency cost, did show group differences, indicating somewhat poorer performance among individuals with AUD.

**Table 2:** Descriptive statistics and group differences on neuropsychological test outcomes (by task) between the sample with AUD and non-clinical reference sample.

Variable	AUD (*n* = 147)	NCS (*n* = 72)	Statistical significance*	Effect size
	*M (SD)*	(*M*, *SD*)	Estimate, standard error, *p*-value (adj)	*(Cohen’s d)*
*SST*				
Stop signal reaction time	194.56 (54.46)	181.86 (46.17)	*b* = 10.20, *se* = 7.55 *p* = .178 (1.00)	0.25 [−0.04, 0.53]
*IST*				
Mean *P* correct at point of decision	0.79 (0.09)	0.80 (0.10)	*b* = -0.01, *se* = 0.01 *p* = .433 (1.00)	−0.09 [−0.37, 0.19]
Sampling errors	2.61 (2.19)	2.33 (1.91)	*b* = 0.40, *se* = 0.31 *p* = .196 (1.00)	0.13 [−0.15, 0.42]
Discrimination errors	1.16 (1.29)	1.42 (1.65)	*b* = -0.32, *se* = 0.21 *p* = .136 (1.00)	−0.18 [−0.47, 0.10]
Mean boxes opened	12.99 (4.55)	13.59 (4.91)	*b* = -0.75, *se* = 0.69 *p* = .280 (1.00)	−0.13 [−0.41, 0.15]
*SWM*				
Between errors	28.88 (18.12)	28.08 (21.91)	*b* = -1.33, *se* = 2.65 *p* = .616 (1.00)	0.04 [−0.24, 0.32]
Strategy	32.58 (6.39)	32.15 (7.28)	*b* = -0.45, *se* = 0.93 *p* = .628 (1.00)	0.06 [−0.22, 0.35]
*IED*				
Total errors	24.67 (23.85)	–	–	–
*AST*				
Switching cost	227.87 (136.12)	–	–	–
Congruency cost	53.33 (44.72)	102.55 (84.53)	*b* = -59.71, *se* = 8.49 ***p* < .001 (*p* < .001)**	−0.81 [−1.11, −0.52]
*CGT*				
Risk adjustment	1.66 (1.09)	1.51 (0.84)	*b* = 0.25, *se* = 0.15 *p* = .089 (1.00)	0.14 [−0.14, 0.43]
Delay aversion	0.19 (0.18)	0.14 (0.15)	*b* = 0.05, *se* = 0.03 *p* = .051 (1.00)	0.29 [0.00, 0.57]
*RVP*				
A’	0.91 (0.05)	0.91 (0.05)	*b* = 0.01, *se* = 0.01 *p* = .442 (1.00)	0.11 [−0.17, 0.39]
Median latency	372.46 (66.77)	378.46 (68.06)	*b* = -16.62, *se* = 9.44 *p* = .079 (1.00)	−0.09 [−0.37, 0.19]
*SOC*				
Problems solved	8.62 (2.10)	8.94 (1.89)	*b* = -0.10, *se* = 0.30 *p* = .732 (1.00)	−0.16 [−0.44, 0.12]

*Note*. NCS = Non-Clinical Reference Sample; SST = Stop Signal Task, IST = Information Sampling Task, SWM = Spatial Working Memory, IED = Intra/Extradimensional Set Shift, AST = Attention Switching Task, CGT = Cambridge Gambling Task, RVP = Rapid Visual Information Processing, SOC = Stockings of Cambridge. Raw *p*-values are presented with adjusted *p*-values in parentheses. *Differences between groups adjusted for age and education level.

Furthermore, neither performance on the SST nor IST at baseline emerged as significant predictors of treatment outcomes (mean weekly alcohol consumption and proportion of days with drinking) among patients with AUD at the 26-, or 12-week follow-up, whilst controlling for covariates (see Tables 3 and 4; results for 12-week follow-up presented in Tables S1-S2 in Supplementary material). The results from the same analyses with imputed data showed congruent results (see Tables S3-S6 in Supplementary material).

**Table 3:** Results from regression models investigating the predictive value of neuropsychological test results on the outcome mean weekly alcohol consumption from Timeline Follow-Back at the 26-week follow-up in the sample with AUD (n = 147).

	*SST* Stop Signal Reaction Time				*IST* Mean *P* correct at point of decision				*IST* Sampling errors				*IST* Discrimination errors				*IST* Mean boxes opened			
Predictor	*b* [95% CI]	*SE*	*P* (adj)	*sr*^*2*^ [95% CI]	*b* [95% CI]	*SE*	*p* (adj)	*sr*^*2*^ [95% CI]	*b* [95% CI]	*SE*	*p* (adj)	*sr*^*2*^ [95% CI]	*b* [95% CI]	*SE*	*p* (adj)	*sr*^*2*^ [95% CI]	*b* [95% CI]	*SE*	*p* (adj)	*sr*^*2*^ [95% CI]
Intercept	3.06 [1.90, 4.60]	0.58	**<.001 (<.001)**		3.41 [1.58, 5.17]	1.07	**.002** (.054)		3.26 [2.28, 4.25]	0.54	**<.001 (<.001)**		3.29 [2.37, 4.35]	0.55	**<.001 (<.001)**		3.40 [2.27, 4.53]	0.68	**<.001 (<.001)**	
CANTAB outcome	0.00 [−0.01, 0.01]	0.00	.283 (1.00)	.01 [.00, .10]	−0.13 [−1.91, 1.85]	1.03	.903 (1.00)	.00 [.00, .03]	0.05 [−0.02, 0.13]	0.04	.222 (1.00)	.01 [.00, .06]	0.00 [−0.17, 0.14]	0.07	.961 (1.00)	.00 [.00, .04]	−0.01 [−0.04, 0.04]	0.02	.788 (1.00)	.00 [.00, .03]
Gender (female)	−0.28 [−0.67, 0.13]	0.19	.156 (1.00)	.01 [.00, .08]	−0.29 [−0.68, 0.12]	0.19	.139 (1.00)	.02 [.00, .10]	−0.30 [−0.69, 0.11]	0.19	.120 (1.00)	.02 [.00, .09]	−0.29 [−0.71, 0.12]	0.19	.140 (1.00)	.02 [.00, .10]	−0.29 [−0.69, 0.10]	0.19	.139 (1.00)	.02 [.00, .09]
Age	−0.01 [−0.03, 0.01]	0.01	.378 (1.00)	.01 [.00, .05]	−0.01 [−0.02, 0.01]	0.01	.519 (1.00)	.00 [.00, .05]	−0.01 [−0.03, 0.01]	0.01	.483 (1.00)	.00 [.00, .05]	−0.01 [−0.02, 0.01]	0.01	.526 (1.00)	.00 [.00, .04]	−0.01 [−0.02, 0.01]	0.01	.504 (1.00)	.00 [.00, .05]
Baseline alcohol consumption	0.03 [0.02, 0.05]	0.01	**<.001 (<.001)**	.14 [.03, .27]	0.03 [0.02, 0.05]	0.01	**<.001 (<.001)**	.14 [.03, .28]	0.03 [0.02, 0.05]	0.01	**<.001 (<.001)**	.14 [.03, .27]	0.03 [0.01, 0.05]	0.01	**<.001 (<.001)**	.14 [.02, .29]	0.03 [0.02, 0.05]	0.01	**<.001 (<.001)**	.14 [.02, .28]
Baseline ASRS	−0.00 [−0.02, 0.02]	0.01	.875 (1.00)	.00 [.00, .04]	−0.00 [−0.02, 0.02]	0.01	.882 (1.00)	.00 [.00, .04]	−0.00 [−0.02, 0.02]	0.01	.665 (1.00)	.00 [.00, .04]	−0.00 [−0.02, 0.02]	0.01	.903 (1.00)	.00 [.00, .04]	−0.00 [−0.02, 0.02]	0.01	.856 (1.00)	.00 [.00, .04]
Treatment arm	−0.10 [−0.24, 0.40]	0.19	.602 (1.00)	.00 [.00, .03]	−0.07 [−0.31, 0.46]	0.19	.719 (1.00)	.00 [.00, .05]	0.06 [−0.32, 0.44]	0.18	.744 (1.00)	.00 [.00, .04]	−0.07 [−0.28, 0.44]	0.19	.719 (1.00)	.00 [.00, .04]	−0.07 [−0.33, 0.44]	0.19	.718 (1.00)	.00 [.00, .04]

*Note*. *b* represents unstandardized regression weights. *sr*^*2*^ represents the semi-partial correlation squared. Raw *p*-values are presented with adjusted *p*-values in parentheses. All confidence intervals were bootstrapped using 1000 samples.

**Table 4:** Results from regression models investigating the predictive value of neuropsychological test results on the outcome proportion of days with drinking from Timeline Follow-Back at the 26-week follow-up in the sample with AUD (n = 147).

	*SST* Stop Signal Reaction Time				*IST* Mean *P* correct at point of decision				*IST* Sampling errors				*IST* Discrimination errors				*IST* Mean boxes opened			
Predictor	*b* [95% CI]	*SE*	*p* (adj)	*sr*^*2*^ [95% CI]	*b* [95% CI]	*SE*	*p* (adj)	*sr*^*2*^ [95% CI]	*b* [95% CI]	*SE*	*p* (adj)	*sr*^*2*^ [95% CI]	*b* [95% CI]	*SE*	*p* (adj)	*sr*^*2*^ [95% CI]	*b* [95% CI]	*SE*	*p* (adj)	*sr*^*2*^ [95% CI]
Intercept	0.13 [0.01, 0.24]	0.07	.053 (1.00)		−0.00 [−0.24, 0.22]	0.12	.977 (1.00)		0.10 [−0.01, 0.20]	0.06	.114 (1.00)		0.09 [−0.00, 0.20]	0.06	.132 (1.00)		0.05 [−0.07, 0.18]	0.07	.504 (1.00)	
CANTAB outcome	−0.00 [−0.00, 0.00]	0.00	.152 (1.00)	.01 [.00, .06]	0.11 [−0.14, 0.34]	0.11	.346 (1.00)	.01 [.00, .06]	−0.01 [−0.02, 0.00]	0.00	.168 (1.00)	.01 [.00, .09]	−0.00 [−0.02, 0.02]	0.01	.805 (1.00)	.00 [.00, .05]	0.00 [−0.00, 0.01]	0.00	.329 (1.00)	.01 [.00, .06]
Gender (female)	0.08 [0.05, 0.13]	0.02	**<.001 (.004)**	.10 [.03, .21]	0.09 [0.05, 0.13]	0.02	**<.001 (.003)**	.11 [.03, .21]	0.09 [0.05, 0.12]	0.02	**<.001 (.003)**	.11 [.03, .21]	0.09 [0.05, 0.13]	0.02	**<.001 (.003)**	.11 [.03, .21]	0.09 [0.05, 0.13]	0.02	**<.001 (.003)**	.11 [.03, .21]
Age	0.00 [0.00, 0.00]	0.00	**.008** (.222)	.05 [.00, .13]	0.00 [0.00, 0.00]	0.00	**.014** (.343)	.04 [.00, .13]	0.00 [0.00, 0.00]	0.00	**.015** (.361)	.04 [.00, .12]	0.00 [0.00, 0.00]	0.00	**.018** (.441)	.04 [.00, .11]	0.00 [0.00, 0.00]	0.00	**.013** (.341)	.04 [.00, .12]
Baseline alcohol consumption	0.06 [−0.05, 0.16]	0.05	.237 (1.00)	.01 [.00, .07]	0.06 [−0.04, 0.17]	0.05	.231 (1.00)	.01 [.00, .07]	0.06 [−0.04, 0.17]	0.05	.203 (1.00)	.01 [.00, .07]	0.06 [−0.04, 0.17]	0.05	.214 (1.00)	.01 [.00, .07]	0.06 [−0.05, 0.17]	0.05	.220 (1.00)	.01 [.00, .06]
Baseline ASRS	−0.00 [−0.00, 0.00]	0.00	.748 (1.00)	.00 [.00, .03]	−0.00 [−0.00, 0.00]	0.00	.880 (1.00)	.00 [.00, .03]	0.00 [−0.00, 0.00]	0.00	.999 (1.00)	.00 [.00, .03]	−0.00 [−0.00, 0.00]	0.00	.702 (1.00)	.00 [.00, .04]	−0.00 [−0.00, 0.00]	0.00	.894 (1.00)	.00 [.00, .03]
Treatment arm	0.00 [−0.04, 0.04]	0.02	.990 (1.00)	.00 [.00, .03]	0.00 [−0.03, 0.05]	0.02	.815 (1.00)	.00 [.00, .03]	0.01 [−0.03, 0.04]	0.02	.785 (1.00)	.00 [.00, .03]	0.00 [−0.04, 0.04]	0.02	.820 (1.00)	.00 [.00, .03]	0.00 [−0.03, 0.05]	0.02	.821 (1.00)	.00 [.00, .03]

*Note*. *b* represents unstandardized regression weights. *sr*^*2*^ represents the semi-partial correlation squared. Raw *p*-values are presented with adjusted *p*-values in parentheses. All confidence intervals were bootstrapped using 1000 samples.

The exploratory analyses did not suggest a predictive value of cognitive performance (SST and IST at baseline) on the additional outcomes of CDT and AUDIT total score measured at the 12-week follow-up (see S7-S8 Tables in Supplementary material).

## Discussion

The main findings in the current study were that the treatment-seeking individuals with moderate AUD, and low levels of psychiatric comorbidities had equal performances in tests of executive function as compared to a non-clinical reference sample. Secondly, performance in specific EFs, as measured with gold-standard computerized tasks SST and IST (CANTAB^®^), were not predictors of treatment outcomes for patients with AUD that had a treatment goal of controlled drinking.

Previous research has suggested impaired cognitive function to be a hallmark of addiction, and that EF is specifically affected in AUD [3,5,9,19,21]. The current study did not corroborate the relationship between impaired EF and AUD, which shows that the clinical populations with AUD may be more heterogeneous in terms of level of cognitive dysfunction. The reasons behind the lack of significant differences in EF, as compared to non-clinical individuals, may be due to the specific AUD sample characteristics. It is suggested that cognitive deficits in AUD result from a combination of heavy use of alcohol over time, and individual vulnerability to certain types of brain damage [37]. In the current study sample, the mean weekly alcohol consumption was lower compared to other clinical samples with AUD included in previous large sample clinical trials [38,39]. Furthermore, the patients in the present study had no documented experience of recent detoxification or inpatient treatment, which is commonly the clinical profile of patients in previous studies of EF in AUD populations [3,9].

Another characteristic of the current sample was the relatively low level of previous treatment (30%). Similar findings were observed in a study including only treatment naïve alcohol-dependent individuals (*n* = 55) who underwent a wide range of cognitive tests specifically sensitive to alcohol use [21]. In the study by Smith et al, no differences were detected in the sample with AUD when compared to healthy controls regarding EF performance. Previous treatment for AUD is known to be a clinical characteristic in individuals with AUD and impaired EF [21]. In these cases, having previous attempts with treatment can be expected to be a proxy for the severity of AUD, as treatment-seeking is significantly higher in more severe AUD [40].

Further, the current sample of individuals reported sub-threshold symptom levels of both anxiety- and depressive symptoms and had a low level of psychiatric comorbid diagnoses. The level of psychiatric comorbidity was significantly different compared to previous studies in the field, which have shown strong associations between severity of AUD and comorbid psychiatric conditions, as well as an association between the severity of AUD and EF [3,9,21]. Another specific characteristic of the patient sample was their level of education. A total of 68% of the included patients with AUD study had a formal educational level corresponding to university-level studies, which was a significantly higher proportion compared to the non-clinical reference sample. Greater academic achievement is often associated with higher general intelligence, which means that the current sample of patients with AUD could be expected to perform well on tests of cognitive abilities [41]. This level of academic performance is also comparably high in comparison to clinical samples included in previous research. For instance, in a study by Lawrence and colleagues, the patients with AUD who had impaired functions in EF had an average of 12 years of education [20]. This is in clear contrast to the current AUD sample. An assumingly high level of premorbid general intelligence may hence have contributed to equal performances in cognitive testing compared to the non-clinical sample.

There may be other reasons to the observed lack of difference in EF between the patients with AUD and the non-clinical sample. The sample with AUD was mainly recruited from a clinic targeting treatment naïve patients, with no obvious impairment in social functioning, or need of social services. This meant that the site of recruitment and the focus on controlled drinking in the original study (the parent RCT trial investigating the efficacy of BSCT vs MET), may have resulted in a rather homogeneous sample [22]. It is also noteworthy that the selection criteria for the trial excluded individuals with a recent history of intoxication, inpatient treatment, or severe health risks related to continuous alcohol consumption, such as impaired liver function. Taken together, these conditions may have attenuated the association between the measured variables. A secondary aim was to investigate whether measures of EF predicted either a reduction in mean weekly alcohol consumption or the proportion of days with drinking in individuals with AUD. There is evidence showing that cognitive function, and specifically measures of EF such as delay discounting and response inhibition, are predictive of clinical outcomes and treatment retention among patients with SUD [4,12,13,16,17]. Similar to the first aim, the limited ability of the EF measures to forecast treatment outcome in the current AUD sample, may reflect differences in disease burden relative to the samples used in the aforementioned studies.. Taken together, the current study showed several of the clinical characteristics (e.g., being treatment naïve, having a higher educational level, and no psychiatric comorbidity) in patients with AUD may be associated with better resilience and more intact cognitive functioning. Further studies are needed to corroborate these findings in other clinical samples.

The findings from the current study have several clinical implications. Interventions, including for example cognitive testing or working memory training, may not be clinically meaningful in individuals with AUD, of moderate severity, high education level and low level of psychiatric co-morbidity. Instead, these patients may indeed be well suited to receive behavioral treatments which require an ability to concentrate, plan and work independently with treatment goals and homework assignments. Interestingly, the patient characteristics in the current sample may be suggested to represent the majority of individuals with AUD in the population, being moderately dependent, with an even gender distribution, and with low levels of psychiatric comorbidity [42,43]. This suggests that impairments in EF in AUD may be more varied than previously suggested. Still, more research is warranted to corroborate the current findings.

### Limitations

There are important limitations to the current study that need mentioning. First, data loss was substantial due to premature stopping of cognitive assessments at baseline in the RCT due to COVID-19. This resulted in a lower level of statistical power, albeit with a fairly large sample size compared to previous research. To handle this limitation, we performed multiple imputations as a robust procedure to be able to perform analyses with the full sample size and increase the statistical power of detecting effects. Second, the study collected detailed baseline data on patients on diagnostics, current alcohol consumption, and related consequences, but did not include any assessment regarding patients’ reports on lifetime consumption levels. This information would have been valuable to the analysis of the validity of the null findings on cognitive impairments. Third, the sample of non-clinical individuals was recruited in the context of another study with the research group, which limited the degree of matched similarity with regards to educational level. Despite this limitation, we found it to be of significance to investigate potential differences between our sample with AUD and a non-clinical sample, given the difficulty to obtain Swedish normative data on the chosen tests. Although participants were not matched on an individual level, the reference sample was found sufficiently similar regarding other relevant characteristics such as gender distribution and age. In addition, we controlled for educational level and age statistically in the analyses. The chosen outcomes in the predictor analyses were weekly alcohol consumption, days with drinking, and the biomarker CDT. One limitation of the study may have been that the sensitivity of the chosen outcomes was insufficient to produce any associations between the CANTAB^®^ tests and the change in alcohol consumption. One measure that may have had limited sensitivity is the AUDIT, which is based on individuals’ self-reported alcohol-related problems over the past year. As a result, some participants may have reported fairly similar levels of alcohol-related problems at the 12-week follow-up as compared to baseline, despite potential changes in their drinking behavior during the study period. Still, the parent study RCT demonstrated clinically relevant pre-post effects on the AUDIT at 26 weeks, indicating that this limitation has not affected the results in a significant way [22]. Further, the chosen biomarker CDT, has been suggested to be less sensitive than PEth to capture changes in alcohol consumption. Still, the fact that an objective marker of alcohol consumption as outcome did not corroborate the association between the tests of EF, and change in alcohol consumption, improves the strength of the current results. Also, the involvement of an objective marker balanced the risk of social desirability bias in self-reports, such as underreporting alcohol consumption at the end of a trial due to a desire to present outcomes that align with treatment success. Lastly, neuropsychological testing of EF was performed with the gold standard battery CANTAB^®^. However, it is not known if the current results would have been different if employing other measures of cognitive functioning.

## Conclusions

The current study demonstrated that a group consisting mainly of treatment-naïve individuals diagnosed with AUD at inclusion, and with a low degree of psychiatric comorbidity, did not exhibit impairments in executive function (EF) as measured by cognitive testing, compared to a non-clinical reference sample. These findings are of particular interest, as previous research on the association between EF and AUD has consistently shown that individuals with AUD often exhibit significant impairments in EF. Results from the main RCT, from which the present patient sample was drawn, showed a substantial reduction in alcohol consumption, and approximately half of the participants achieved a low-risk drinking level (with the goal of controlled drinking) after receiving short-term treatment [22]. Given these clinical findings, behavioral treatments that are cognitively demanding can be expected to be feasible and successful for individuals with AUD, with corresponding clinical characteristics to this study sample. More research is warranted, to better understand the role of cognitive function in AUD by also increasing diversity among the recruited individuals in future research. This means for example including participants with varying educational levels, socioeconomic status, and wider age spans. Of relevance would be the need to evaluate changes in cognitive function before and after treatment to be able to detect both subtle and obvious differences over time and their differential impact on clinical outcomes.

## Supporting information

S1 Table Results from regression models investigating the predictive value of neuropsychological test results on the outcome mean weekly alcohol consumption from Timeline Follow-Back at the 12-week follow-up in the sample with AUD. (DOCX)

S2 Table Results from regression models investigating the predictive value of neuropsychological test results on the outcome proportion of days with drinking from Timeline Follow-Back at the 12-week follow-up in the sample with AUD. (DOCX)

S3 Table Results from regression models investigating the predictive value of neuropsychological test results on the outcome mean weekly alcohol consumption from Timeline Follow-Back at the 26-week follow-up in the sample with AUD (with imputed data). (DOCX)

S4 Table Results from regression models investigating the predictive value of neuropsychological test results on the outcome proportion of days with drinking from Timeline Follow-Back at the 26-week follow-up in the sample with AUD. (DOCX)

S5 Table Results from regression models investigating the predictive value of neuropsychological test results on the outcome mean weekly alcohol consumption from Timeline Follow-Back at the 12-week follow-up in the sample with AUD (with imputed data). (DOCX)

S6 Table Results from regression models investigating the predictive value of neuropsychological test results on the outcome proportion of days with drinking from Timeline Follow-Back at the 12-week follow-up in the sample with AUD (with imputed data). (DOCX)

S7 Table Results from regression models investigating the predictive value of neuropsychological test results on the outcome Carbohydrate Deficient Transferrin the 12-week follow-up in the sample with AUD. (DOCX)

S8 Table Results from regression models investigating the predictive value of neuropsychological test results on the outcome Alcohol Use Disorders Identification Test total score at the 12-week follow-up in the sample with AUD. (DOCX)
