# Supplementary material for: Executive function in alcohol use disorder with low psychiatric comorbidity: Comparison with a non-clinical sample and predictive value for treatment outcome
Source: PLoS One. 2026 Sep 11;21(9):e0350955. doi: 10.1371/journal.pone.0350955 (PMC13567833; doi:10.1371/journal.pone.0350955)
Supplement: Supplementary file 1 [file pone.0350955.s001.docx]

Supplementary material for

Executive Function in Alcohol Use Disorder with Low Psychiatric Comorbidity: Comparison with a Non-clinical Sample and Predictive Value for Treatment Outcome

Stina Ingesson-Hammarberg^1*^, Maria Å. Garke^1^, Lotfi Khemiri^1, 2^, Anders Hammarberg^1^, Nitya Jayaram-Lindström^1^

^1^ Centre for Psychiatry Research, Department of Clinical Neuroscience, Karolinska Institutet, & Stockholm Health Care Services, Region Stockholm, Sweden.

^2^ Department of Medical Epidemiology and Biostatistics, Karolinska Institutet, Stockholm, Sweden

* Corresponding author: Stina Ingesson-Hammarberg, stina.ingesson@ki.se

**Information on R packages used for analysis**

Packages included tidyverse for data handling (Wickham et al., 2019), psych for descriptive statistics (Revelle, 2024), and mice for data imputation (van Buuren & Groothuis-Oudshoorn, 2011).

Wickham H, Averick M, Bryan J, Chang W, McGowan LD, François R, Grolemund G, Hayes A, Henry L, Hester J, Kuhn M, Pedersen TL, Miller E, Bache SM, Müller K, Ooms J, Robinson D, Seidel DP, Spinu V, Takahashi K, Vaughan D, Wilke C, Woo K, Yutani H (2019). “Welcome to the tidyverse.”. Journal of Open Source Software, 4(43), 1686. doi:10.21105/joss.01686 <https://doi.org/10.21105/joss.01686>.

William Revelle (2026). psych: Procedures for Psychological, Psychometric, and Personality Research. Northwestern University, Evanston, Illinois. R package version 2.6.3, https://CRAN.R-project.org/package=psych.

Stef van Buuren, Karin Groothuis-Oudshoorn (2011). mice: Multivariate Imputation by Chained Equations in R. Journal of Statistical Software, 45(3), 1-67. DOI 10.18637/jss.v045.i03.

**Table S1***.* **Results from regression models investigating the predictive value of neuropsychological test results on the outcome mean weekly alcohol consumption from Timeline Follow-Back at the 12-week follow-up in the sample with AUD (n = 147)**

|  | *SST*  Stop Signal Reaction Time | | | | *IST*  Mean *P* correct at point of decision | | | | *IST*  Sampling errors | | | | *IST*  Discrimination errors | | | | *IST*  Mean boxes opened | | | |
| --- | --- | --- | --- | --- | --- | --- | --- | --- | --- | --- | --- | --- | --- | --- | --- | --- | --- | --- | --- | --- |
| **Predictor** | *b* [95% CI] | *SE* | *p*  (adj) | *sr^2^* [95% CI] | *b* [95% CI] | *SE* | *p*  (adj) | *sr^2^* [95% CI] | *b* [95% CI] | *SE* | *p* (adj) | *sr^2^* [95% CI] | *b* [95% CI] | *SE* | *p*  (adj) | *sr^2^* [95% CI] | *b* [95% CI] | *SE* | *p*  (adj) | *sr^2^* [95% CI] |
| Intercept | 3.02 [1.95, 4.29] | 0.56 | **<.001 (<.001)** |  | 2.95 [1.10, 4.56] | 1.02 | **.005** (.125) |  | 2.98 [2.08, 3.90] | 0.52 | **<.001 (<.001)** |  | 3.03 [2.12, 3.92] | 0.52 | **<.001 (<.001)** |  | 3.09 [1.94, 4.15] | 0.63 | **<.001 (<.001)** |  |
| CANTAB outcome | 0.00 [-0.01, 0.00] | 0.00 | .979 (1.00) | .00 [.00, .05] | 0.08 [-1.62, 1.83] | 1.00 | .934 (1.00) | .00 [.00, .02] | 0.04 [-0.04, 0.12] | 0.04 | .309 (1.00) | .01 [.00, .04] | -0.00 [-0.13, 0.11] | 0.07 | .961 (1.00) | .00 [.00, .02] | -0.00 [-0.04, 0.03] | 0.02 | .867 (1.00) | .00 [.00, .03] |
| Gender (female) | -0.15 [-0.50, 0.19] | 0.19 | .427 (1.00) | .00 [.00, .04] | -0.16 [-0.50, 0.20] | 0.19 | .423 (1.00) | .00 [.00, .04] | -0.15 [-0.50, 0.19] | 0.19 | .435 (1.00) | .00 [.00, .04] | -0.15 [-0.48, 0.20] | 0.19 | .426 (1.00) | .00 [.00, .04] | -0.15 [-0.51, 0.22] | 0.19 | .430 (1.00) | .00 [.00, .04] |
| Age | -0.00 [-0.02, 0.02] | 0.01 | .761 (1.00) | .00 [.00, .03] | -0.00 [-0.02, 0.01] | 0.01 | .768 (1.00) | .00 [.00, .03] | -0.00 [-0.02, 0.01] | 0.01 | .720 (1.00) | .00 [.00, .03] | -0.00 [-0.02, 0.02] | 0.01 | .767 (1.00) | .00 [.00, .03] | -0.00 [-0.02, 0.01] | 0.01 | .744 (1.00) | .00 [.00, .03] |
| Baseline alcohol consumption | 0.04 [0.02, 0.06] | 0.01 | **<.001 (<.001)** | .20 [.07, .34] | 0.04 [0.03, 0.06] | 0.01 | **<.001 (<.001)** | .20 [.07, .35] | 0.04 [0.03, 0.06] | 0.01 | **<.001 (<.001)** | .20 [.07, .33] | 0.04 [0.03, 0.06] | 0.01 | **<.001 (<.001)** | .20 [.07, .35] | 0.04  [0.03, 0.06] | 0.01 | **<.001 (<.001)** | .20 [.07, .34] |
| Baseline ASRS | -0.01 [-0.03, 0.00] | 0.01 | .153 (1.00) | .01 [.00, .06] | -0.01 [-0.03, 0.00] | 0.01 | .164 (1.00) | .01 [.00, .05] | -0.01 [-0.03, 0.00] | 0.01 | .106 (1.00) | .02 [.00, .07] | -0.01 [-0.03, 0.00] | 0.01 | .152 (1.00) | .01 [.00, .06] | -0.01 [-0.03, 0.00] | 0.01 | .151 (1.00) | .01 [.00, .06] |
| Treatment arm | -0.11 [-0.45, 0.27] | 0.18 | .557 (1.00) | .00 [.00, .03] | -0.11 [-0.47, 0.24] | 0.18 | .552 (1.00) | .00 [.00, .04] | -0.12 [-0.47, 0.24] | 0.18 | .511 (1.00) | .00 [.00, .04] | -0.11 [-0.46, 0.25] | 0.18 | .552 (1.00) | .00 [.00, .03] | -0.11 [-0.45, 0.24] | 0.18 | .552 (1.00) | .00 [.00, .03] |
| *Note*. *b* represents unstandardized regression weights. *sr^2^* represents the semi-partial correlation squared. Raw *p*-values are presented with adjusted *p*-values in parentheses. All confidence intervals were bootstrapped using 1000 samples. | | | | | | | | | | | | | | | | | | | | |
