# Supplementary material for: Executive function in alcohol use disorder with low psychiatric comorbidity: Comparison with a non-clinical sample and predictive value for treatment outcome
Source: PLoS One. 2026 Sep 11;21(9):e0350955. doi: 10.1371/journal.pone.0350955 (PMC13567833; doi:10.1371/journal.pone.0350955)
Supplement: Supplementary file 2 [file pone.0350955.s002.docx]

**Table S2. Results from regression models investigating the predictive value of neuropsychological test results on the outcome proportion of days with drinking from Timeline Follow-Back at the 12-week follow-up in the sample with AUD (n = 147)**

|  | *SST*  Stop Signal Reaction Time | | | | *IST*  Mean *P* correct at point of decision | | | | *IST*  Sampling errors | | | | *IST*  Discrimination errors | | | | *IST*  Mean boxes opened | | | |
| --- | --- | --- | --- | --- | --- | --- | --- | --- | --- | --- | --- | --- | --- | --- | --- | --- | --- | --- | --- | --- |
| **Predictor** | *b* [95% CI] | *SE* | *p* (adj) | *sr^2^* [95% CI] | *b* [95% CI] | *SE* | *p* (adj) | *sr^2^* [95% CI] | *b* [95% CI] | *SE* | *p* (adj) | *sr^2^* [95% CI] | *b* [95% CI] | *SE* | *p*  (adj) | *sr^2^* [95% CI] | *b* [95% CI] | *SE* | *p* (adj) | *sr^2^* [95% CI] |
| Intercept | -0.01 [-0.20, 0.23] | 0.10 | .914 (1.00) |  | -0.02 [-0.31, 0.27] | 0.18 | .906 (1.00) |  | 0.02 [-0.14, 0.17] | 0.09 | .810 (1.00) |  | 0.03 [-0.13, 0.18] | 0.09 | .764 (1.00) |  | 0.01 [-0.16, 0.20] | 0.11 | .915 (1.00) |  |
| CANTAB outcome | 0.00 [-0.00, 0.00] | 0.00 | .375 (1.00) | .00 [.00, .05] | 0.05 [-0.26, 0.36] | 0.18 | .764 (1.00) | .00 [.00, .02] | 0.00 [-0.01, 0.02] | 0.01 | .750 (1.00) | .00 [.00, .02] | -0.01 [-0.03, 0.02] | 0.01 | .650 (1.00) | .00 [.00, .02] | 0.00 [-0.01, 0.01] | 0.00 | .835 (1.00) | .00 [.00, .02] |
| Gender (female) | 0.00 [-0.06, 0.07] | 0.03 | .977 (1.00) | .00 [.00, .02] | -0.00 [-0.07, 0.07] | 0.03 | .967 (1.00) | .00 [.00, .02] | -0.00 [-0.06, 0.06] | 0.03 | .980 (1.00) | .00 [.00, .02] | 0.00 [-0.06, 0.07] | 0.03 | .996 (1.00) | .00 [.00, .02] | -0.00 [-0.07, 0.07] | 0.03 | .963 (1.00) | .00 [.00, .02] |
| Age | 0.00 [-0.00, 0.00] | 0.00 | .512 (1.00) | .00 [.00, .02] | 0.00 [-0.00, 0.00] | 0.00 | .337 (1.00) | .00 [.00, .03] | 0.00 [-0.00, 0.00] | 0.00 | .361 (1.00) | .00 [.00, .03] | 0.00 [-0.00, 0.00] | 0.00 | .322 (1.00) | .00 [.00, .04] | 0.00 [-0.00, 0.00] | 0.00 | .343 (1.00) | .00 [.00, .03] |
| Baseline alcohol consumption | 0.67 [0.52, 0.80] | 0.08 | **<.001 (<.001)** | .30 [.17, .42] | 0.66 [0.51, 0.82] | 0.08 | **<.001 (<.001)** | .30 [.17, .42] | 0.66 [0.51, 0.81] | 0.08 | **<.001 (<.001)** | .30 [.17, .42] | 0.66 [0.50, 0.81] | 0.08 | **<.001 (<.001)** | .29 [.17, .40] | 0.66 [0.51, 0.81] | 0.08 | **<.001 (<.001)** | .30 [.17, .41] |
| Baseline ASRS | -0.00 [-0.01, -0.00] | 0.00 | .087 (1.00) | .01 [.00, .05] | -0.00 [-0.00, 0.00] | 0.00 | .115 (1.00) | .01 [.00, .05] | -0.00 [-0.01, -0.00] | 0.00 | .091 (1.00) | .01 [.00, .05] | -0.00 [-0.01, 0.00] | 0.00 | .093 (1.00) | .01 [.00, .05] | -0.00 [-0.01, -0.00] | 0.00 | .111 (1.00) | .01 [.00, .05] |
| Treatment arm | 0.00 [-0.06, 0.07] | 0.03 | .943 (1.00) | .00 [.00, .02] | -0.00 [-0.07, 0.06] | 0.03 | .970 (1.00) | .00 [.00, .02] | -0.00 [-0.06, 0.06] | 0.03 | .952 (1.00) | .00 [.00, .02] | -0.00 [-0.06, 0.06] | 0.03 | .967 (1.00) | .00 [.00, .02] | -0.00 [-0.07, 0.05] | 0.03 | .967 (1.00) | .00 [.00, .02] |
| *Note*. *b* represents unstandardized regression weights. *sr^2^* represents the semi-partial correlation squared. Raw *p*-values are presented with adjusted *p*-values in parentheses. All confidence intervals were bootstrapped using 1000 samples. | | | | | | | | | | | | | | | | | | | | |
