# Supplementary material for: Executive function in alcohol use disorder with low psychiatric comorbidity: Comparison with a non-clinical sample and predictive value for treatment outcome
Source: PLoS One. 2026 Sep 11;21(9):e0350955. doi: 10.1371/journal.pone.0350955 (PMC13567833; doi:10.1371/journal.pone.0350955)
Supplement: Supplementary file 3 [file pone.0350955.s003.docx]

**Table S3. Results from regression models investigating the predictive value of neuropsychological test results on the outcome mean weekly alcohol consumption from Timeline Follow-Back at the 26-week follow-up in the sample with AUD (with imputed data, n = 250)**

|  | *SST*  Stop Signal Reaction Time | | | *IST*  Mean *P* correct at point of decision | | | *IST*  Sampling errors | | | *IST*  Discrimination errors | | | *IST*  Mean boxes opened | | |
| --- | --- | --- | --- | --- | --- | --- | --- | --- | --- | --- | --- | --- | --- | --- | --- |
| **Predictor** | *b* | *SE* | *p* | *B* | *SE* | *p* | *b* | *SE* | *p* | *B* | *SE* | *p* | *b* | *SE* | *p* |
| Intercept | 2.71 | 0.50 | **<.001** | 2.59 | 1.05 | .019 | 3.00 | 0.47 | **<.001** | 3.03 | 0.49 | **<.001** | 3.00 | 0.59 | **<.001** |
| CANTAB outcome | 0.00 | 0.00 | .256 | 0.52 | 1.11 | .646 | 0.01 | 0.06 | .896 | -0.03 | 0.07 | .680 | 0.00 | 0.02 | .971 |
| Gender (female) | -0.31 | 0.16 | .057 | -0.32 | 0.16 | .049 | -0.32 | 0.16 | .048 | -0.31 | 0.16 | .057 | -0.32 | 0.16 | .049 |
| Age | -0.00 | 0.01 | .690 | -0.00 | 0.01 | .877 | -0.00 | 0.01 | .868 | -0.00 | 0.01 | .907 | -0.00 | 0.01 | .871 |
| Baseline alcohol consumption | 0.03 | 0.01 | **<.001** | 0.03 | 0.01 | **<.001** | 0.03 | 0.01 | **<.001** | 0.03 | 0.01 | **<.001** | 0.03 | 0.01 | **<.001** |
| Baseline ASRS | 0.00 | 0.01 | .866 | 0.00 | 0.01 | .863 | 0.00 | 0.01 | .925 | 0.00 | 0.01 | .959 | 0.00 | 0.01 | .897 |
| Treatment arm | 0.21 | 0.15 | .168 | 0.19 | 0.15 | .214 | 0.19 | 0.15 | .213 | 0.20 | 0.16 | .208 | 0.19 | 0.16 | .215 |
| *Note*. *b* represents unstandardized regression weights. Raw *p*-values are presented. | | | | | | | | | | | | | | | |
