# Supplementary material for: Executive function in alcohol use disorder with low psychiatric comorbidity: Comparison with a non-clinical sample and predictive value for treatment outcome
Source: PLoS One. 2026 Sep 11;21(9):e0350955. doi: 10.1371/journal.pone.0350955 (PMC13567833; doi:10.1371/journal.pone.0350955)
Supplement: Supplementary file 4 [file pone.0350955.s004.docx]

**Table S4. Results from regression models investigating the predictive value of neuropsychological test results on the outcome proportion of days with drinking from Timeline Follow-Back at the 26-week follow-up in the sample with AUD (with imputed data, n = 250)**

|  | *SST*  Stop Signal Reaction Time | | | *IST*  Mean *P* correct at point of decision | | | *IST*  Sampling errors | | | *IST*  Discrimination errors | | | *IST*  Mean boxes opened | | |
| --- | --- | --- | --- | --- | --- | --- | --- | --- | --- | --- | --- | --- | --- | --- | --- |
| **Predictor** | *b* | *SE* | *p* | *B* | *SE* | *p* | *b* | *SE* | *p* | *b* | *SE* | *p* | *b* | *SE* | *p* |
| Intercept | 0.39 | 0.06 | **<.001** | 0.36 | 0.12 | .005 | 0.41 | 0.05 | **<.001** | 0.38 | 0.05 | **<.001** | 0.39 | 0.07 | **<.001** |
| CANTAB outcome | 0.00 | 0.00 | .968 | 0.04 | 0.13 | .739 | -0.01 | 0.00 | .104 | 0.01 | 0.01 | .200 | 0.00 | 0.00 | .950 |
| Gender (female) | 0.08 | 0.02 | **<.001** | 0.08 | 0.02 | **<.001** | 0.08 | 0.02 | **<.001** | 0.08 | 0.02 | **<.001** | 0.08 | 0.02 | **<.001** |
| Age | 0.00 | 0.00 | .088 | 0.00 | 0.00 | .073 | 0.00 | 0.00 | .082 | 0.00 | 0.00 | .090 | 0.00 | 0.00 | .072 |
| Baseline alcohol consumption | 0.05 | 0.04 | .216 | 0.05 | 0.04 | .211 | 0.06 | 0.04 | .162 | 0.06 | 0.04 | .173 | 0.05 | 0.04 | .208 |
| Baseline ASRS | -0.00 | 0.00 | .429 | -0.00 | 0.00 | .447 | -0.00 | 0.00 | .631 | -0.00 | 0.00 | .558 | -0.00 | 0.00 | .436 |
| Treatment arm | -0.03 | 0.02 | .054 | -0.03 | 0.02 | .053 | -0.03 | 0.02 | .063 | -0.03 | 0.02 | .049 | -0.03 | 0.02 | .054 |
| *Note*. *B* represents unstandardized regression weights. Raw *p*-values are presented. | | | | | | | | | | | | | | | |
