# Supplementary material for: Executive function in alcohol use disorder with low psychiatric comorbidity: Comparison with a non-clinical sample and predictive value for treatment outcome
Source: PLoS One. 2026 Sep 11;21(9):e0350955. doi: 10.1371/journal.pone.0350955 (PMC13567833; doi:10.1371/journal.pone.0350955)
Supplement: Supplementary file 5 [file pone.0350955.s005.docx]

**Table S5. Results from regression models investigating the predictive value of neuropsychological test results on the outcome mean weekly alcohol consumption from Timeline Follow-Back at the 12-week follow-up in the sample with AUD (with imputed data, n = 250)**

|  | *SST*  Stop Signal Reaction Time | | | *IST*  Mean *P* correct at point of decision | | | *IST*  Sampling errors | | | *IST*  Discrimination errors | | | *IST*  Mean boxes opened | | |
| --- | --- | --- | --- | --- | --- | --- | --- | --- | --- | --- | --- | --- | --- | --- | --- |
| **Predictor** | *b* | *SE* | *p* | *B* | *SE* | *P* | *b* | *SE* | *p* | *b* | *SE* | *p* | *b* | *SE* | *p* |
| Intercept | 2.74 | 0.46 | **<.001** | 2.33 | 0.80 | .004 | 2.68 | 0.42 | **<.001** | 2.68 | 0.41 | **<.001** | 2.69 | 0.49 | **<.001** |
| CANTAB outcome | -0.00 | 0.00 | .848 | 0.46 | 0.82 | .577 | 0.02 | 0.04 | .703 | 0.02 | 0.06 | .798 | 0.00 | 0.02 | .963 |
| Gender (female) | -0.15 | 0.14 | .304 | -0.14 | 0.14 | .310 | -0.14 | 0.14 | .305 | -0.15 | 0.14 | .303 | -0.15 | 0.14 | .306 |
| Age | -0.00 | 0.01 | .999 | -0.00 | 0.01 | .972 | -0.00 | 0.01 | .975 | -0.00 | 0.01 | .961 | -0.00 | 0.01 | .963 |
| Baseline alcohol consumption | 0.04 | 0.01 | **<.001** | 0.04 | 0.01 | **<.001** | 0.04 | 0.01 | **<.001** | 0.04 | 0.01 | **<.001** | 0.04 | 0.01 | **<.001** |
| Baseline ASRS | -0.01 | 0.01 | .260 | -0.01 | 0.01 | .280 | -0.01 | 0.01 | .231 | -0.01 | 0.01 | .277 | -0.01 | 0.01 | .261 |
| Treatment arm | 0.16 | 0.14 | .243 | 0.16 | 0.14 | .239 | 0.16 | 0.14 | .245 | 0.16 | 0.14 | .232 | 0.16 | 0.14 | .236 |
| *Note*. *b* represents unstandardized regression weights. Raw *p*-values are presented. | | | | | | | | | | | | | | | |
