# Supplementary material for: Executive function in alcohol use disorder with low psychiatric comorbidity: Comparison with a non-clinical sample and predictive value for treatment outcome
Source: PLoS One. 2026 Sep 11;21(9):e0350955. doi: 10.1371/journal.pone.0350955 (PMC13567833; doi:10.1371/journal.pone.0350955)
Supplement: Supplementary file 6 [file pone.0350955.s006.docx]

**Table S6. Results from regression models investigating the predictive value of neuropsychological test results on the outcome proportion of days with drinking from Timeline Follow-Back at the 12-week follow-up in the sample with AUD (with imputed data, n = 250)**

|  | *SST*  Stop Signal Reaction Time | | | *IST*  Mean *P* correct at point of decision | | | *IST*  Sampling errors | | | *IST*  Discrimination errors | | | *IST*  Mean boxes opened | | |
| --- | --- | --- | --- | --- | --- | --- | --- | --- | --- | --- | --- | --- | --- | --- | --- |
| **Predictor** | *b* | *SE* | *p* | *B* | *SE* | *p* | *b* | *SE* | *p* | *b* | *SE* | *p* | *b* | *SE* | *p* |
| Intercept | 0.31 | 0.07 | **<.001** | 0.29 | 0.12 | .016 | 0.35 | 0.06 | **<.001** | 0.34 | 0.06 | **<.001** | 0.33 | 0.07 | **<.001** |
| CANTAB outcome | 0.00 | 0.00 | .411 | 0.07 | 0.12 | .557 | -0.00 | 0.00 | .670 | 0.00 | 0.01 | .756 | 0.00 | 0.00 | .798 |
| Gender (female) | 0.02 | 0.02 | .371 | 0.02 | 0.02 | .374 | 0.02 | 0.02 | .381 | 0.02 | 0.02 | .417 | 0.02 | 0.02 | .385 |
| Age | 0.00 | 0.00 | .818 | 0.00 | 0.00 | .651 | 0.00 | 0.00 | .672 | 0.00 | 0.00 | .668 | 0.00 | 0.00 | .656 |
| Baseline alcohol consumption | 0.48 | 0.05 | **<.001** | 0.48 | 0.05 | **<.001** | 0.48 | 0.05 | **<.001** | 0.48 | 0.05 | **<.001** | 0.48 | 0.05 | **<.001** |
| Baseline ASRS | -0.00 | 0.00 | .144 | -0.00 | 0.00 | .151 | -0.00 | 0.00 | .165 | -0.00 | 0.00 | .160 | -0.00 | 0.00 | .145 |
| Treatment arm | 0.02 | 0.02 | .242 | 0.02 | 0.02 | .278 | 0.02 | 0.02 | .270 | 0.02 | 0.02 | .269 | 0.02 | 0.02 | .280 |
| *Note*. *b* represents unstandardized regression weights. Raw *p*-values are presented. | | | | | | | | | | | | | | | |
