# Supplementary material for: Executive function in alcohol use disorder with low psychiatric comorbidity: Comparison with a non-clinical sample and predictive value for treatment outcome
Source: PLoS One. 2026 Sep 11;21(9):e0350955. doi: 10.1371/journal.pone.0350955 (PMC13567833; doi:10.1371/journal.pone.0350955)
Supplement: Supplementary file 7 [file pone.0350955.s007.docx]

**Table S7. Results from regression models investigating the predictive value of neuropsychological test results on the outcome Carbohydrate Deficient Transferrin the 12-week follow-up in the sample with AUD (n = 94)**

|  | *SST*  Stop Signal Reaction Time | | | | *IST*  Mean *P* correct at point of decision | | | | *IST*  Sampling errors | | | | *IST*  Discrimination errors | | | | *IST*  Mean boxes opened | | | |
| --- | --- | --- | --- | --- | --- | --- | --- | --- | --- | --- | --- | --- | --- | --- | --- | --- | --- | --- | --- | --- |
| **Predictor** | *b* [95% CI] | *SE* | *p* | *sr^2^* [95% CI] | *b* [95% CI] | *SE* | *p* | *sr^2^* [95% CI] | *b* [95% CI] | *SE* | *p* | *sr^2^* [95% CI] | *b* [95% CI] | *SE* | *P* | *sr^2^* [95% CI] | *b* [95% CI] | *SE* | *p* | *sr^2^* [95% CI] |
| Intercept | 1.01 [0.75, 1.27] | 0.13 | **<.001** |  | 1.02 [0.61, 1.36] | 0.21 | **<.001** |  | 0.98 [0.76, 1.20] | 0.11 | **<.001** |  | 0.97 [0.74, 1.17] | 0.11 | **<.001** |  | 1.00 [0.72, 1.26] | 0.14 | **<.001** |  |
| CANTAB outcome | -0.00 [-0.00, 0.00] | 0.00 | .614 | .00 [.00, .02] | -0.04 [-0.37, 0.38] | 0.20 | .825 | .00 [.00, .02] | 0.00 [-0.01, 0.02] | 0.01 | .645 | .00 [.00, .02] | 0.01 [-0.02, 0.05] | 0.01 | .449 | .00 [.00, .03] | -0.00 [-0.01, 0.01] | 0.00 | .790 | .00 [.00, .02] |
| Gender (female) | -0.09 [-0.16, -0.02] | 0.04 | .019 | .02 [.00, .06] | -0.09 [-0.17, -0.02] | 0.04 | .021 | .02 [.00, .07] | -0.09 [-0.16, -0.02] | 0.04 | .021 | .02 [.00, .07] | -0.09 [-0.16, -0.02] | 0.04 | .016 | .02 [.00, .07] | -0.09 [-0.16, -0.02] | 0.04 | .022 | .02 [.00, .06] |
| Age | 0.00 [-0.00, 0.00] | 0.00 | .972 | .00 [.00, .02] | -0.00 [-0.00, 0.00] | 0.00 | .924 | .00 [.00, .02] | -0.00 [-0.00, 0.00] | 0.00 | .933 | .00 [.00, .03] | -0.00 [-0.00, 0.00] | 0.00 | .911 | .00 [.00, .02] | -0.00 [-0.00, 0.00] | 0.00 | .911 | .00 [.00, .02] |
| Baseline alcohol consumption | 0.17 [0.14, 0.23] | 0.02 | **<.001** | .50 [.34, .62] | 0.17 [0.15, 0.24] | 0.02 | **<.001** | .52 [.33, .65] | 0.17 [0.14, 0.23] | 0.02 | **<.001** | .52 [.34, .65] | 0.17 [0.14, 0.24] | 0.02 | **<.001** | .51 [.31, .65] | 0.17 [0.15, 0.24] | 0.02 | **<.001** | .52 [.33, .65] |
| Baseline ASRS | -0.00 [-0.00, 0.00] | 0.00 | .434 | .00 [.00, .02] | -0.00 [-0.00, 0.00] | 0.00 | .429 | .00 [.00, .02] | -0.00 [-0.00, 0.00] | 0.00 | .387 | .00 [.00, .02] | -0.00 [-0.00, 0.00] | 0.00 | .513 | .00 [.00, .02] | -0.00 [-0.00, 0.00] | 0.00 | .421 | .00 [.00, .02] |
| Treatment arm | 0.14 [0.07, 0.21] | 0.04 | **<.001** | .06 [.01, .13] | 0.14 [0.08, 0.21] | 0.04 | **<.001** | .06 [.02, .14] | 0.14 [0.07, 0.21] | 0.04 | **<.001** | .06 [.01, .14] | 0.14 [0.08, 0.21] | 0.04 | **<.001** | .06 [.01, .14] | 0.14 [0.07, 0.21] | 0.04 | **<.001** | .06 [.01, .13] |
| *Note*. *B* represents unstandardized regression weights. *Sr^2^* represents the semi-partial correlation squared. Raw *p*-values are presented with adjusted *p*-values in parentheses. All confidence intervals were bootstrapped using 1000 samples. | | | | | | | | | | | | | | | | | | | | |
