# Supplementary material for: Executive function in alcohol use disorder with low psychiatric comorbidity: Comparison with a non-clinical sample and predictive value for treatment outcome
Source: PLoS One. 2026 Sep 11;21(9):e0350955. doi: 10.1371/journal.pone.0350955 (PMC13567833; doi:10.1371/journal.pone.0350955)
Supplement: Supplementary file 8 [file pone.0350955.s008.docx]

**Table S8. Results from regression models investigating the predictive value of neuropsychological test results on the outcome Alcohol Use Disorders Identification Test total score at the 12-week follow-up in the sample with AUD (n = 125)**

|  | *SST*  Stop Signal Reaction Time | | | | *IST*  Mean *P* correct at point of decision | | | | *IST*  Sampling errors | | | | *IST*  Discrimination errors | | | | *IST*  Mean boxes opened | | | |
| --- | --- | --- | --- | --- | --- | --- | --- | --- | --- | --- | --- | --- | --- | --- | --- | --- | --- | --- | --- | --- |
| **Predictor** | *b* [95% CI] | *SE* | *p* | *sr^2^* [95% CI] | *b* [95% CI] | *SE* | *p* | *sr^2^* [95% CI] | *b* [95% CI] | *SE* | *p* | *sr^2^* [95% CI] | *b* [95% CI] | *SE* | *p* | *sr^2^* [95% CI] | *b* [95% CI] | *SE* | *p* | *sr^2^* [95% CI] |
| Intercept | 10.86 [5.84, 15.56] | 2.59 | **<.001** |  | 14.05 [6.45, 21.39] | 3.94 | **<.001** |  | 10.17 [5.19, 14.82] | 2.51 | **<.001** |  | 10.26 [5.44, 15.17] | 2.48 | **<.001** |  | 11.75 [6.15, 16.93] | 2.79 | **<.001** |  |
| CANTAB outcome | -0.00 [-0.02, 0.01] | 0.01 | .509 | .00 [.00, .02] | -4.42 [-10.89, 1.76] | 3.69 | .233 | .00 [.00, .03] | 0.09 [-0.20, 0.39] | 0.16 | .589 | .00 [.00, .02] | 0.28 [-0.16, 0.67] | 0.27 | .295 | .00 [.00, .02] | -0.08 [-0.22, 0.06] | 0.07 | .282 | .00 [.00, .03] |
| Gender (female) | -1.53 [-2.95, -0.34] | 0.71 | .034 | .02 [.00, .05] | -1.46 [-2.84, -0.13] | 0.71 | .042 | .01 [.00, .05] | -1.48 [-2.78, -0.18] | 0.71 | .040 | .01 [.00, .05] | -1.57 [-3.01, -0.29] | 0.71 | .030 | .02 [.00, .06] | -1.46 [-2.87, -0.21] | 0.71 | .043 | .01 [.00, .05] |
| Age | -0.10 [-0.16, -0.03] | 0.03 | .003 | .03 [.00, .08] | -0.11 [-0.16, -0.05] | 0.03 | .001 | .04 [.01, .09] | -0.10 [-0.16, -0.04] | 0.03 | .001 | .04 [.01, .09] | -0.11 [-0.17, -0.05] | 0.03 | .001 | .04 [.01, .09] | -0.11 [-0.16, -0.05] | 0.03 | **<.001** | .04 [.01, .09] |
| Baseline alcohol consumption | 0.55 [0.39, 0.69] | 0.07 | **<.001** | .23 [.12, .33] | 0.55 [0.39, 0.70] | 0.07 | **<.001** | .23 [.12, .35] | 0.55 [0.38, 0.69] | 0.07 | **<.001** | .23 [.11, .34] | 0.54 [0.38, 0.69] | 0.07 | **<.001** | .23 [.11, .34] | 0.55 [0.40, 0.69] | 0.07 | **<.001** | .23 [.12, .34] |
| Baseline ASRS | 0.05 [-0.01, 0.11] | 0.03 | .135 | .01 [.00, .04] | 0.04 [-0.01, 0.10] | 0.03 | .211 | .01 [.00, .03] | 0.04 [-0.01, 0.10] | 0.03 | .194 | .01 [.00, .03] | 0.05 [-0.01, 0.10] | 0.03 | .114 | .01 [.00, .04] | 0.04 [-0.02, 0.10] | 0.03 | .206 | .01 [.00, .03] |
| Treatment arm | -0.98 [-2.24, 0.29] | 0.67 | .149 | .01 [.00, .03] | -0.95 [-2.18, 0.30] | 0.67 | .156 | .01 [.00, .03] | -0.95 [-2.24, 0.29] | 0.67 | .158 | .01 [.00, .04] | -0.95 [-2.20, 0.27] | 0.67 | .157 | .01 [.00, .03] | -0.94 [-2.22, 0.27] | 0.67 | .164 | .01 [.00, .03] |
| *Note*. *b* represents unstandardized regression weights. *sr^2^* represents the semi-partial correlation squared. Raw *p*-values are presented with adjusted *p*-values in parentheses. All confidence intervals were bootstrapped using 1000 samples. | | | | | | | | | | | | | | | | | | | | |
